# Disability-Related Costs of Children with Disabilities in the Philippines

**DOI:** 10.3390/ijerph20136304

**Published:** 2023-07-06

**Authors:** Ludovico Carraro, Alex Robinson, Bilal Hakeem, Abner Manlapaz, Rosela Agcaoili

**Affiliations:** 1 Independent Consultant, Oxford OX3 0JE, UK; 2 Nossal Institute for Global Health, University of Melbourne, Melbourne, VIC 3010, Australia; 3 Oxford Policy Management, Oxford OX1 3HJ, UK; bilal.hakeem@opml.co.uk; 4 Life Haven Center for Independent Living, Valenzuela City 1442, Philippines; abner.manlapaz@gmail.com; 5 United Nations Children’s Fund (UNICEF), Mandaluyong City 1550, Philippines; ragcaoili@unicef.org

**Keywords:** disability, children, extra costs, standard of living, Philippines

## Abstract

The assessment of disability-related costs among children remains a largely under-researched subject with related questions rarely included in surveys. This paper addresses this issue through a unique mixed methods study conducted in the Philippines combining a nationally representative survey and in-depth interviews with families and health professionals. To quantify the extra costs associated with disabilities, the research used the standard of living approach, whereby expenditure levels of families with children with and without disabilities were compared in relation to different measures of living standards. The results find consistent evidence of high extra costs among households that have children with disabilities and point to health expenses as the leading source. Using an asset index as the indicator of living standards, a child with a disability is estimated to require between 40% and 80% extra expenditure to reach the same living standard of other children. However, the size of extra costs is substantially higher when the measure of the standard of living relies on a broader set of deprivations. In such cases, higher estimates of extra costs are likely to be the result of the lack of an inclusive environment. Critically, this points to the need to provide not only financial support but also inclusive services, especially in health and education.

## 1. Introduction

This paper reports findings from a mixed methods study to calculate the additional costs experienced by households with children with disabilities in the Philippines. That disability and poverty are inherently linked has long been assumed. The argument is bidirectional: if you are poor, you are more likely to have a disability and if you have a disability, you are more likely to be poor [1]. However, evidence to support this argument has often been anecdotal or contrary [2]. For example, a comparison focusing only on monetary poverty between people with and without disabilities in Nepal and Vietnam found no significant differences between the two groups [3]. Analyses of income and consumption expenditure to identify the additional costs experienced by households with persons with disabilities have also been inconclusive [4]. The situation is compounded by non-standardized approaches to defining and measuring disability and a lack of robust disability data at national levels, including for the measurement of childhood disability [5,6].

Increasingly, the standard of living (SOL) approach has been used to provide a more complete estimation of the additional costs experienced by households with persons with disabilities. This draws on Amartya Sen’s concept of the ‘conversion gap’, which explains why persons with disabilities may incur higher expenses to achieve the same SOL outcomes as persons without disabilities [7]. That is, a household with a person with disabilities and a household without a person with disabilities with the same income level and similar characteristics, such as location and household size, should have a similar standard of living. If the two households do not, the difference is attributable to disability-related costs [8].

The SOL approach has been used to understand disability-related costs in Cambodia, China, Ghana, Turkey, the United Kingdom (UK), the United States, and Vietnam [9,10,11,12,13,14,15,16]. Extra costs associated with disability have been found to vary by severity of impairment, employment status, and gender [3]. Higher extra costs associated with disabilities have been identified in urban areas compared to rural areas [10]. Estimates from Ghana placed the extra costs of a disability for a household at 26% with increased poverty of between 39% and 53% [11]. For Cambodia, the poverty rate rose from 18% to 37% for households with persons with disabilities with only 7% of disability-related costs met after accounting for government support [9]. In Turkey, disability-related expenses accounted for up to 23% of household incomes [12]. UK government transfer payments fell behind rising disability-related costs for households between 2013 and 2016 [14]. In Kenya, increased parenting stress was identified among poor households with a family member with disabilities [17].

There are three key limitations of the SOL approach [8,18]. Firstly, the approach often uses available datasets that rarely include disability-related expenditure, for example, spending on assistive devices and their maintenance, or are not fully representative of persons with disabilities in a population [5,9]. The use of household asset indexes and subjective observations of living conditions can also be problematic. Secondly, the approach is an indirect method, and while it estimates the overall extra cost, it does not identify the specific items that make up those extra costs [9]. Consequently, the approach provides limited detail to inform policy decisions. Thirdly, there is a risk of underestimating disability-related costs by assuming living standards can be improved through higher disability-related expenditure alone. The SOL approach does not account for barriers to participation in a social life or to accessing services, including a lack of services, limited knowledge of services that may be available, an inability to pay, or the indirect costs associated with disability, such as foregone income [19].

The lack of robust cost data in the Philippines has been a barrier to disability-related policy development, including the establishment of social protection policies targeting households with children with disabilities. In response, this study was commissioned by the Department of Social Welfare and Development (DSWD), Government of the Philippines, in partnership with the United Nations Children’s Fund (UNICEF) with support from Australia’s Department of Foreign Affairs and Trade (DFAT).

This study contributes an SOL-informed assessment of the additional costs experienced by households of children with disabilities in comparison to households of children without disabilities. This is supplemented by qualitative findings to clarify gaps on barriers, cost items, and indirect costs associated with disability [2,9,19]. This study is unique to the Philippines but contributes to the wider evidence base on SOL, poverty, and disability. Human ethics approval for this study was obtained from the University of Melbourne’s School of Population and Global Health Human Ethics Advisory Group (No. 2021-21437-20298-4).

## 2. Materials and Methods

### 2.1. Guiding Principles

Disability is complex and contested. In considering disability, this study drew on the United Nations Convention on the Rights of Persons with Disabilities (CRPD) description of disability:

*Persons with disabilities include those who have long-term physical, mental, intellectual or sensory impairments which in interaction with various barriers may hinder their full and effective participation in society on an equal basis with others*.

[20]

Households with children with disabilities were identified via government disability identification (ID) card lists and by the use of question sets developed by the Washington Group on Disability Statistics (see Section 2.2 and Section 2.4). Children were defined as persons under 18 years of age [21].

**Figure 1:**
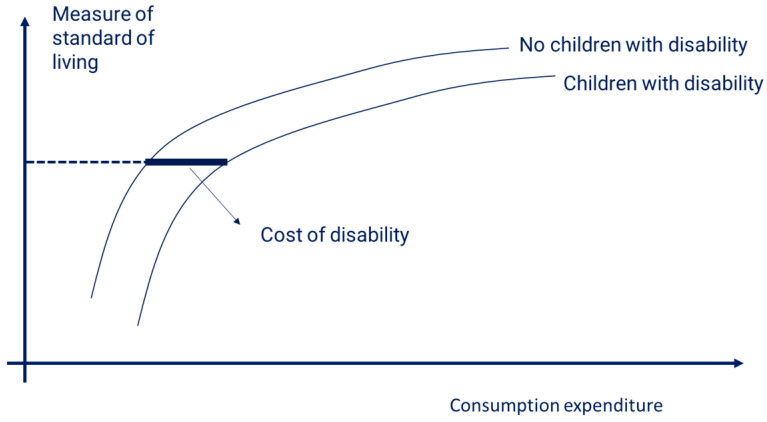
Standard of living and the cost of disability. Source: Authors’ graphical representation.

The SOL understanding that underpins this study is shown in Figure 1 and follows two assumptions [22]. Firstly, we expected a positive relationship between standard of living and consumption expenditure. That is, the higher the expenditure, the higher the standard of living achieved. Secondly, the extra cost associated with disabilities was assessed in relation to a control group of households with children without disabilities. This allowed costs to be compared between households with and without children with disabilities at the same welfare level.

The approach involved an assessment of extra costs within the constraints of what money can buy in the Philippines and the compromises every household must make when faced with budget constraints. What money can buy depends not only on the availability of services and their accessibility but also on prevailing attitudes, behaviors, and social norms. For example, a household with a child with disabilities might not have costs associated with formal education if there is no school providing the support and assistance required for her/his attendance. Or a child might not be attending school because stigma or bullying makes attendance impossible. Even with a higher income, a family may face difficulties or may not be able to access services for their child.

In applying Figure 1, we did not make an assumption on how the level of extra cost changes as we move from low to high living standards (regardless of what is depicted in Figure 1). We did not assume that the costs of disability are constant for different living standards: they could be decreasing or increasing. Furthermore, costs may vary depending on the severity or type of disability.

### 2.2. Household Survey Design

The household survey was designed to collect information across four pillars of child rights derived from the UN Convention on the Rights of the Child with a focus on the first two pillars presented below [23]:

- The right to survival, including adequate nutrition, healthcare, water and sanitation, and a safe place to live.
- The right to development, which includes access to quality education.
- The right to protection, including the safety of the child within the home and community.
- The right to participation, including participation by the child in daily activities.

The household survey included questions on household characteristics and composition and primary activities, such as employment or education, income and transfers, and household expenditure. Questions on access to services were included as were questions on the use of disability identification (ID) cards, attendance at special education schools or classes, and the use of assistive technology.

The Washington Group Short Set of questions on functioning were asked of all adults in respondent households [24]. The two Washington Group/UNICEF Child Functioning Modules were used according to the age of the child, that is, either 2 to 4 years or 5 to 17 years [25]. The Short Set and Child Functioning Modules were asked of all households regardless of whether a child in the household had a disability ID card (see Section 2.4). In each location, key informant interviews were completed with officials at the municipality and barangay levels on the availability of services and the prices of sample items were collected from local stores.

The household survey was delayed due to COVID-19 restrictions with data collection completed between November 2021 and June 2022.

### 2.3. Sampling Frame

The household survey used two samples of households in the same geographical area. The first was a nationally representative sample of households of children who have a disability ID card. Disability ID card lists were provided by Local Government Units (LGUs) and formed the basis of the sampling frame with children with disabilities selected from the lists by systematic random sampling. Sampling weights were used to estimate the overall number of children with a disability ID card in the Philippines. This should not be confused with all children with disabilities in the country. Survey locations (barangays) were selected randomly by probability proportional to population size in four regions (strata): Luzon, Visayas, Mindanao, and the National Capital Region.

The second sample was households with children with no disability ID selected from the same area or block by systematic random sampling. This formed the comparison group for this study. This comparison sample was not representative of all children without a disability ID card in the Philippines as the sample was not drawn from a full list of households in the survey locations. Instead, the comparison group was drawn from a smaller list of eligible households residing in the same block or area as selected children with a disability ID card. Within this second group, we also found children with disabilities based on responses to the Child Functioning Modules.

The same survey questions were asked of all households. In total, 2753 interviews were fully completed with households of children with and without a disability ID card in 240 locations. This included all 17 regions with data collection in 69 of the Philippine’s 82 provinces.

### 2.4. Disability Measures

We used different proxies for disability. These were the disability ID card to establish the sampling frame and the Washington Group question sets. Disability ID cards are issued by LGUs in response to registration requests by individuals and, reflective of the ‘individual’ or ‘medical’ model of disability, include impairment types or health conditions [26]. The disability ID cards have a concessionary benefit that allows a holder to pay for certain goods and services at a discounted price. A card may be issued following direct observation by an LGU official for an ‘apparent’ disability or following the issuance of a certificate by a medical expert for a ‘non-apparent’ disability [27]. The administration of disability ID cards was found to be inconsistent with variations in categorization and the impairments and conditions listed. In contrast, the Washington Group question sets draw on the social model of disability and CRPD understandings (see Section 2.1) and use activity limitations to capture functioning difficulties as a proxy for disability. The Washington Group questions were developed to provide a standardized and comparable measure of disability for use in censuses and surveys.

As noted, the Washington Short Set of questions were asked of adults. The relevant Child Functioning Module questions were asked of the child’s main carer. In terms of Washington Group question responses, we considered children with disabilities to be all children for whom the carer reports at least ‘some difficulty’. As standard, the responses across the different functional domains were ‘no difficulty’, ‘some difficulty’, ‘a lot of difficulty’, and ‘cannot do at all’. While it is usual practice to use the ‘a lot of difficulty’ cutoff to indicate disability in censuses and surveys, many carers of children with a disability ID card reported the child experienced ‘some difficulty’ only. In analysis, we separated cases where the difficulty is reported as ‘some difficulty’, signaling a milder disability, and others where the disability is likely to be more severe (a lot of difficulty or cannot do at all).

Sampling children with a disability ID card could potentially create bias if families who apply for the card have particular socio-economic characteristics. We found some evidence that households with an ID card were better off (more likely to be in the highest consumption quintile) and the parents better educated. However, the differences were not large and households in the bottom quintile were not under-represented. These differences were further reduced by use of the Washington Group question responses.

### 2.5. Calculation of Extra Costs and Equivalence Scales

We calculated extra costs using a regression model of non-monetary measures of standard of living over consumption expenditure (as a proxy for income), a measure of disability, and other control variables. Control variables included the geographical location, the age of the household head, and the size/type of households. The general regression model was as follows:

(1) $$\mathrm{SOL}=\beta_{0\;}+\beta_{1}\times \ln\left(cons\right)+\beta_{2}\times dis+\beta_{i}\times HHtype_{i}+\beta_{j}\times control\;variable_{j}+\varepsilon$$

where “SOL” was the standard of living measure. “ln(*cons*)” was the transformation of the household consumption expenditure in logarithmic terms. “*dis*” stood for the disability measure, for example, whether there was a child in the household with a reported functional difficulty. “*HHtype*” values were dummy variables capturing the different household sizes/compositions. Introducing dummy variables for household types rather than the household size allowed for non-linearity in measuring the effect of an extra household member on the SOL and was substantially equivalent to controlling for the effect of the change in household size on the SOL.

We used two non-monetary measures of the SOL: household asset indexes and measures of non-deprivation. The asset index included both dwelling characteristics (quality of walls, access to drinking water, sanitation, size of dwelling, and tenure) and assets (ownership of car, motorbike, stove, fridge, personal computer, television, and smart phone). It was estimated using polychoric principal component analysis, which is theoretically superior to simple principal component analysis when there are many categorical variables [28]. The values of the coefficients of the asset index are reported in Appendix A.

The measure of non-deprivation was based on indicators used in the multi-dimensional poverty measure developed by the Philippine Statistics Authority [29]. This includes indicators in four main dimensions: health and nutrition, housing, education, and employment. Employment indicators were excluded for use with children, and for education we only included non-enrollment among children aged 5 to 17, thus excluding adults’ educational attainment. For nutrition, indicators used by the Philippine Statistics Authority include food expenditure; however, we used non-monetary indicators of food security as consumption expenditure is one of our main explanatory variables. Specifically, the indicators used covered lack of adequate access to sanitation, water, shelter, tenure, electricity, information and communication technologies, education, and food security measures, in line with the World Food Programme’s food consumption score [30]. The overall indicator used counted the number of non-deprivations.

The regression modeled the SOL, but in measuring the monetary cost of disability we were ultimately interested in the relationship between the coefficients of the consumption expenditure variable and the measure of disability [19,22]. After testing different functional forms for consumption expenditure (quadratic and squared root), the best fit was given by a logarithmic functional form: higher levels of consumption expenditure have diminishing returns on the SOL. This implies that the cost of disability is not constant across different SOL levels but increases proportionally with the level of expenditure.

The cost of disability at the household level was computed by assuming that households with and without disability reach the same SOL ($\left(\mathrm{SOL}|dis=0\right)=\left(\mathrm{SOL}|dis=1\right))$ and had the same attributes, so the following applies:

(2) $$\beta_{1}ln\left(cons_{d}\right)+\beta_{2}=\beta_{1}ln\left(cons_{nd}\right)$$

where $\beta_{2}$ was the coefficient of the dummy capturing the presence of disability, $\beta_{1}$ was the coefficient of the logarithm of consumption expenditure estimated in Equation (1), and $cons_{d}$.and $cons_{nd}$ represented the level of consumption expenditure of the household with and without disability, respectively. Therefore, the average disability cost at the household level could be expressed as the ratio of consumption expenditure and represents the equivalence scale as follows:

(3) $$EqScale=e^{-\frac{\beta_{2}}{\beta_{1}}}$$

The above expression computed an average equivalence scale of disability at the household level. To estimate the equivalence scale of an individual child with disabilities, the expression would require some indirect imputation. The assessment of the extra cost of disability at an individual child level is relevant for social welfare policy development and is also necessary for the correct assessment of the implications of disability-related costs on poverty measurement. In countries and datasets where there are often single-member households, the regression could be estimated within this specific group and Equation (3) would provide the equivalence scale of the person with a disability. This approach has been used in the UK and in European Union countries where the SOL equation has been estimated for single-person households and couples or focused on households composed exclusively of persons with disabilities [13,22,31]. As our interest was children with disabilities who live in households with at least one adult, it was not possible to estimate the regression on single-member households. Instead, assuming a per capita approach in moving from household- to person-level consumption expenditure, the equivalence scale of a person with disabilities could be computed as follows:

(4) $$Person\;level\;extra\;cost=\frac{household\;level\;extra\;cost}{\left(average\;number\;of\;persons\;with\;disabilities\;in\;household/average\;household\;size\right)}$$

For example, a household-level extra cost of 10% where there is one household member with disabilities and a household size of four members implies a disability extra cost of 40% for the household member with disabilities.

### 2.6. Qualitative Data Collection

An induction visit included consultations with key stakeholders and home visits to families of children with disabilities in Metro Manila and surrounds. The finalization of the sampling frame was supported by interviews with government officials and representatives of organizations of persons with disabilities.

Preliminary semi-structured interviews informed the household survey design, including the refinement of the cost categories. Participants were a convenience sample of seven households with children with disabilities and a representative of a parents’ association identified through the Life Haven Centre for Independent Living’s networks. Due to COVID-19 restrictions, preliminary interviews were conducted via video call. Interview participants included households with more than one child with disabilities and households located outside of Manila. Interview topics were expenses incurred; hypothetical costs, including for assistive products or specialized health services; opportunity costs of care, including forgone income; social participation by carers; and the child’s opportunities for play and social interaction.

Following the completion of the household survey, in-person interviews were completed with twenty-nine households with children with disabilities. Interviews were not completed with households of children without disabilities. Participants were purposively selected from survey respondents to explore unmet needs in areas emerging from a preliminary analysis of the household survey data. Topic areas included foregone health treatment, assistive products, home adaptations and accessibility, non-enrollment in school, and the support needs of parent or primary carer. These post-survey interviews provided additional context and informed the interpretation of the findings. Interviews were conducted in-person following the lifting of COVID-19 restrictions. Interview questions were not specific to the COVID-19 period and covered the period from the birth of the child to the time of the interview. Specific questions on the impacts of COVID-19 were also included.

Supplementary interviews with eight allied health professionals were completed to understand potential unmet needs and related costs for different impairment types. These interviews addressed the issue that families of children with disabilities often had a low awareness of health service availability and were not able to estimate health-related costs based on their child’s particular needs. The professions represented included audiologists, neurologists, occupational therapists, speech therapists, orthotists, and physiotherapists. Estimates of costs by these professionals were based on health conditions most frequently seen by the professional and the ideal available treatment from birth to adulthood.

### 2.7. Qualitative Data Analysis

Interview transcripts were transcribed and translated into the English language from Tagalog and/or local languages. Transcripts were inputted into NVivo software for coding and thematic analysis. A sample of transcripts was coded by two researchers and compared to produce a common code list. Initial deductive analysis was guided by the research objectives and interview guide themes. Sub-themes were then identified and the themes were adjusted as needed. Differences in interpretation were resolved by logical reasoning and discussion. No major differences of interpretation were noted during analysis.

Responses from allied health professionals were transcribed into a standard Excel format. Cost data were used to produce composite stories to illustrate potential costs associated with different health conditions. Final stories were validated by a research colleague, who is a practicing pediatric occupational therapist, and project stakeholders in the Philippines.

## 3. Results

### 3.1. Descriptive Statistics

Table 1 reports the distribution of household types separating households with at least one child with disabilities and households with children without disabilities. The table also shows the average number of children with disabilities in households with at least one child with disabilities. Nuclear families, or households composed exclusively of parents and children, represented almost two thirds of all households with children. Other households were three-generation households, with grandparents, and complex household types, for example, where the parents’ siblings were present or the parents of at least one of the children were not in the household. There were no large differences between household types with or without children with disabilities; however, there tended to be more children within nuclear family households with children with disabilities. The average number of children with disabilities in the household was 1.16.

Table 2 shows basic descriptive statistics for households with and without children with disabilities and separates nuclear families from other types of households. All the main variables used in the analysis are included. These were the consumption expenditure in logarithm terms, the age of the household head, the household size, the location of the household, and the two variables chosen to capture the living standards. These were the asset index and the number of deprivations.

### 3.2. Regression Results

Several regressions were estimated, restricting the sample to certain household types, using different measures of disability (ownership of disability card and functional difficulty) and different measures of living standards. The results that proved more consistent and where it was easier to derive the extra cost for a child with disabilities came from the sample of nuclear families (two-thirds of our sample) and the use of functional difficulties, which included the advantage of being able to distinguish the severity of the disability by reported difficulty. Using complex household compositions, such as three-generation households, made ascertaining the relationship between the SOL, consumption expenditure, and disability more difficult.

Results of the regressions are reported in Table 3. This includes the estimated implications of the proportional extra expenditure required by a household with a child with a disability to reach the same standard of living as a household without a child with a disability.

In both models, the consumption expenditure was positively correlated with the living standard measure and highly significant. As predicted, the measure of disability had negative coefficients, which are consistently highly significant in the case of moderate/severe functional difficulties and smaller and with a lower degree of accuracy in the case of milder functional difficulties. In terms of other explanatory variables, significant variables were similar across the asset index model and the number of deprivations model.

Estimates of the proportional higher expenditure incurred by families with a child with disabilities differed when we compared results using the asset index coefficients with results based on the number of deprivations. In both models, moderate/severe functional difficulties involved twice as much extra expenditure compared to milder functional difficulties. However, much higher differences in expenditure were obtained using the number of deprivations compared to the asset index.

**Figure 2:**
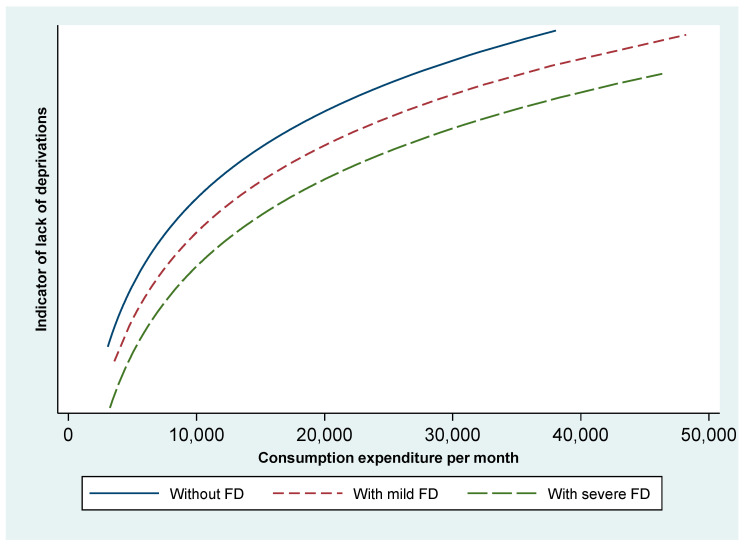
Standard of living and the cost of disability—deprivation model. FD stands for functional difficulties among children. Source: Authors’ graphical representation.

Using the deprivation model, the estimated relationship between consumption expenditure and SOL is represented in Figure 2. This shows that the curves were more concave for families with children with disabilities, meaning that the disability costs increased as we moved from low to high living standards. Further, the cost was higher for severe disabilities. The same trends were observed in the asset index model but with less pronounced differences.

The proportional extra expenditure reported in Table 3 was for an average household. Expressing this in terms of an equivalence scale for the child required making further assumptions. In the Philippines, poverty measures are computed using the per capita income with the income divided by the household size. Therefore, in the case of the asset index model, transforming the household-level extra cost of 19% when there are children with moderate/severe functional difficulties meant the extra expenditure per child with functional difficulties could be estimated by dividing by the ratio of the average number of children with these functional difficulties and the average household size (see Equation (4)). This resulted in an estimate of 80% extra expenditure per child with a disability compared to other household members.

Based on the extra expenditure estimate derived from the model using the asset index as an SOL measure, we can assume the equivalence scale for a child with moderate/severe functional difficulties is 1.8 and for those with mild functional difficulties it is 1.4. Using these equivalence scales in computing an adjusted measure of household needs (modified equivalent household size) and then dividing the consumption expenditure by this adjusted household size results in significantly higher poverty measures for households with children with disabilities. When additional costs were included, poverty estimates for households with children with disabilities increased by 25% and poverty rates were 50% higher when compared to households with children without disabilities.

Analysis of consumption patterns across families with and without children with disabilities clearly showed the main sources of extra costs. The share of household budget spent on health by households with a child with disabilities was three times higher than for households with children without disabilities. One-third of children with disabilities were not enrolled in school. Nevertheless, the share of education expenditure was higher among families with children with disabilities compared to families without children with disabilities. Other costs frequently cited to be higher for people with disabilities are transportation costs. This was clearly identified in qualitative interviews, but significant differences were not found in the quantitative survey. Alongside what may be prohibitive transportation costs resulting in low use by some, the timing of the household survey may have been a contributing factor with restricted movements during lockdowns and school closures.

### 3.3. Disability ID Cards

Our sampling methodology, including the computation of sampling weights using the inverse probability of sampling, enabled us to calculate an overall estimate of the number of children with a disability ID card in the Philippines. This resulted in a mid-estimate of 325,000 children (with a 95% confidence interval of 297,000 and 353,000) with a disability ID card in the country. However, our findings showed that the children most disadvantaged were children with functional difficulties that did not have a disability ID card.

### 3.4. Health Expenditure

As shown from household survey data, the highest costs were related to health. Reported cost items from qualitative interviews included routine and specialist consultations; surgery; maintenance medicines; nutritional supplements; and the sourcing, fitting, and maintenance of assistive devices. These were additional to the routine health costs experienced by children with and without disabilities. The highest reported costs were for specialist diagnoses and surgery at tertiary facilities, with costs in the tens of thousands of (US) dollars reported. Routine check-ups were a significant source of expenditure with frequent visits (up to three times a week) to multiple specialists.

Medications were noted as the highest regular cost for households of children with chronic health conditions, including children with epilepsy, kidney disease, and cerebral palsy. A few respondents could source cheaper medicines, including directly from manufacturers or through contacts with medical professionals or local politicians. Vitamin supplements were commonly prescribed and expensive. Further costs included the purchase of blenders, electricity, and the time and expense of individually prepared food. While an example of the use of feeding tubes was reported, simple assistive products, for example, feeding chairs to support a child while eating, were rarely used.

Overall, the knowledge of assistive products and devices was low. Parents often became aware of assistive products through informal sources or by chance, for example, through seeing another person using a product, including videos on social networking services. Families reported being unable to, or assuming they were unable to, afford assistive products. A family described paying significant optometrist fees and then being unable to afford the prescription glasses. The use of one assistive product did not preclude unmet needs for other products. The appropriateness and safety of existing assistive products was questioned and the costs of maintenance and replacement noted. Information was also scarce. For example, parents of a child who had outgrown her prosthetic leg did not know how to contact the charity that provided it. Buying assistive products from online marketplaces was noted as was the use of secondhand products. The use of homemade assistive products, such as canes and handrails from wood or bamboo, was reported.

### 3.5. Adaptations to Home

Adaptations to homes reduce physical barriers and promote independent the use of the home by the child. Few families had adapted their homes. Adaptations were difficult in rented accommodation as they required the landowner’s consent, and making adaptations was often not prioritized in the face of competing demands for financial resources and time.

Reported adaptations included installing a new toilet, installing a concrete floor to aid mobility, and constructing a sleeping platform. A family reported they were rebuilding their home to provide additional space and privacy for their child. Another example was a family who wanted to install child-safe electrical sockets for their child but had not yet been able to do so. Other adjustments included the purchase of mattress toppers and installation of air conditioning. Another family explained how they kept lights in their house on to assist their child with low vision. The costs of the electrical bills for the air conditioner and lighting examples were considered very high.

### 3.6. Costs of Education and Learning

Not all children were enrolled in school. Among children aged 5 to 17 with a disability ID card and at least some functional difficulty, enrollment was 66%, whereas enrollment among children without a disability was 96%. Additional costs of attending school for children with disabilities included higher fees for some special education (SPED) schools and the need for a shadow or support teacher. Examples of the costs of a shadow teacher needing to be shared between families were reported. Currently, the Philippines does not have a comprehensive system of inclusive education.

Costs of travel were cited as a frequent concern across all domains but particularly for daily school attendance. This included having to travel further to access a school with a SPED program or, simply, to a school that would accept their child. Accompanying a child to and from school increased expenditure on public transport as well as opportunity costs of forgone work with some parents staying at the school throughout the day to support their child. A lack of reasonable accommodation was reported with simple adjustments not implemented, such as children with visual impairments being seated at the back of a class. Administrative barriers to annual reenrollment, such as being asked to provide medical certificates, were noted.

Social costs included bullying by pupils and parents without disabilities, for example, bullying by a parent who could not accept that a child with disabilities could perform better than their child without disabilities. In addition, an incident of physical assault by a teacher on a child with disabilities led to the parents withdrawing their child from school. Limited social interaction and opportunities for play were reported, including parents not allowing their child outside for fear they may become sick. Overall, there was limited expenditure on play items, but the use of downloaded or online games for mobile devices was noted.

During COVID-19, remote learning was not provided by all schools. Families accessing remote learning paid extra for modules and tests. It was noted that the modules could leave a child understimulated and bored. The example of a child with a psychosocial disability attending a private SPED school was noted as the household’s ‘heaviest expense’. The school reduced fees under COVID-19, but any savings were offset by having to buy a laptop and accessories for online learning.

### 3.7. Costs of Care

Costs of care included financial and non-financial costs, including forgone social or economic opportunities. Care was predominantly, but not exclusively, provided by women. Primary and secondary carers included siblings, grandparents, and other family members. Care could be provided to more than one child with disabilities in a household. A total of 24% of households with children with moderate/severe functional difficulties included more than one child with disabilities. Qualitative interview responses shed further light on care. For example, two households had hired an outside carer at one point. In one case the cost had been paid by a relative and in the other the household could not afford to continue paying the carer. Absent parents with no further contact with the child or family were reported. Carers emphasized their role was not ‘babysitting’ and was a full-time support role. Older carers were concerned over the future of the child with disabilities who would survive them.

No carers reported receiving emotional support for themselves or respite care. The need for additional support, such as moving a heavier or older child, was noted. However, one respondent considered it hard to trust others to provide this assistance. Opportunity costs included having to give up work; lost income, such as from selling goods outside of the busiest hours; and moving to lower paid jobs for more flexible work hours. One mother reported having to pay extra for ice to keep her fish fresh for sale while she accompanied her child to school. Work could be missed for extended periods when a child needed health care, particularly for travel to tertiary healthcare facilities. Two people may be needed to assist with moving a child, adding to travel costs and accompanying siblings missing school. Not having relatives in urban areas was considered an additional challenge as it would result in extra costs for accommodation and food when attending tertiary health facilities.

### 3.8. Allied Health Costs

Three composite stories were developed to illustrate potential additional health expenditure from birth to 18 years old. These were based on scenarios of ‘full intervention’, including consultations with multiple specialists; check-ups; medications; and assistive devices with fitting, maintenance, and replacement. The scenarios were based on services available in the Philippines; however, this does not mean all services were available and accessible in all areas. While households often underestimated these potential costs, allied professionals could overestimate in their calculations, including overprescribing the frequency of consultations. As such, the following are high-end cost scenarios.

For a child with spina bifida, the total health cost estimates of a family before adulthood could be in the region of USD 39,000. We also considered multiple impairments. For a child with an intellectual disability, autism, and hearing impairment, the estimates reached USD 46,500. The scenario for a child with cerebral palsy and epilepsy resulted in an estimate of USD 90,000. These estimates were costs to the household and assume no health insurance, deductions, or other financial support. The health expenditure reported in the household survey was based on access to a limited suite of health services and can be assumed to be lower than that required to meet actual unmet health needs.

## 4. Discussion and Limitations

Households with children with disabilities were consistently disadvantaged in comparison to households with children without disabilities. The highest costs were related to health expenditure. Households of children with disabilities spent three times more on health-related goods and services than households without children with disabilities.

The inclusion of disability-related costs resulted in poverty rates that were 25% higher than if disability-related costs were not accounted for. After accounting for disability-related costs, households with children with disabilities had poverty rates that were 50% higher than households of children without disabilities. High monetary costs existed with large unmet needs. Unmet needs and the fact that some needs cannot be addressed by money alone accounted for different cost estimates using the two different SOL indicators. Using the number of deprivations indicated substantial extra costs that cannot be met by money alone. For example, school enrollment rates were considerably lower among children with disabilities than among children without disabilities and particularly low among children with high support needs, that is, children who have severe difficulties with self-care, such as feeding or dressing themselves. Among children with high support needs, school enrollment was only 50% even among households in the top income distribution quintile. The findings show that children with disabilities in high-income households in the Philippines are also deprived of basic rights, such as access to education. Further, an income transfer to households with children with disabilities will not solve this problem alone. This is not specific to the Philippines and has also been found in higher-income settings.

In comparison, the asset index approach is more likely to show the displacement of expenditure towards disability-related costs as it is based on commonly available items. Therefore, the two types of SOL indicators and models have the possibility to complement each other and offer different insights. Further research in other countries could investigate the comparison of SOL estimates of extra costs between different measures based on asset indexes and the number of deprivations. While models using deprivations need further testing, they present the advantage of introducing indicators that can be collected at the individual level, such as school attendance and access to health care [32].

Qualitative findings highlight further barriers beyond direct financial costs that contribute to unmet needs. These include a limited availability of services, imperfect knowledge of potential support for the child, incomplete understandings of the actual needs of the child, stigma, and the challenges of balancing care against the financial and non-financial costs of missed socio-economic opportunities. Access to social networks, better information, and higher incomes could mitigate these issues for some. Irrespective of income, significant challenges were reported by all interview participants.

Supplementing the SOL approach to quantifying extra costs with direct questions on disability-related expenses and unmet needs as well as qualitative interviews provided a greater depth of understanding of the challenges faced by children with disabilities and their families. It is only through this more comprehensive assessment of extra costs and unmet needs that it is possible to develop effective policy solutions. On one hand, the findings of substantial monetary costs justify the creation of social protection instruments, in particular, financial support that can address demand costs. On the other hand, these are clearly not sufficient alone. It is necessary to also ensure specific support through the provision of services, particularly in health and education.

Based on the results of this study, three main recommendations can be made to address and recognize the disability-related costs faced by children with disabilities in the Philippines: financial support through a disability allowance, the expansion of services, and improved poverty measurements. Although there are legislative proposals to the Congress of the Philippines aiming to provide financial assistance to persons with disabilities, the Philippines currently has no standard disability allowance that is implemented across the country. However, there are local examples, such as in the Bangsamoro region, where a monthly stipend of PHP 500 per month has been provided to persons with disabilities who are poor since 2020.

Cash transfers that address at least part of the extra costs of disability are a flexible policy instrument that is likely to have a direct impact on children’s well-being and in reducing financial barriers. In the Philippines, a cash disability allowance would complement the existing discounts provided through the disability ID card, particularly as the concession benefits of the card are unequally distributed. This is particularly pertinent for people living in rural areas and for those who cannot afford to pay the unsubsidized part of the cost of items and services. Such an allowance could also support access to essential services.

The main source of extra disability-related costs is health costs. Expanding the PhilHealth health insurance benefit packages that children with disabilities are entitled to is required. This should be accompanied by extending eligible PhilHealth services, which are currently provided by a limited number of tertiary hospitals in urban areas, to local levels throughout the country. This could be supplemented by improved services and information at the community level, including through community-based rehabilitation and inclusive development initiatives.

Monetary poverty measures in the Philippines should be adapted and account for the extra costs of disabilities. This correction is essential to better assess needs and correct the underestimation of poverty among households that include persons with disabilities. As noted, the correction of monetary poverty indicators alone is insufficient. There is a need to better account for the non-income dimensions of poverty in policy interventions that aim to address inequities experienced by children and adults, with disabilities.

### Study Limitations

Given that having a disability ID card entitles the family to receive certain discounts and subsidies, the consumption expenditure measure was adjusted to reflect actual consumption. The subsidies received based on the self-reported use of the card for different discounts were included. This was an indirect adjustment and might have underestimated the actual cost of disabilities if the real value of the subsidy was not properly reflected. It is important to note that not all households with a card made use of it to obtain discounts and, on average, the value of the subsidy was a small percentage of households’ overall consumption at 2.5%.

This study used consumption expenditure as a proxy of income, but we did not consider how consumption was financed. When faced with high health expenditure, households may need to borrow money and become indebted. This, in turn, impacts on the living conditions and health and well-being of the household. How these dynamics impact households of children with disabilities was not covered in this study and warrants further research.

There are limited data options to ensure a nationally representative sample of persons with disabilities. The disability ID card was the preferred option as it is a national scheme and allows the identification of individuals. However, the disability ID card, including how the eligibility for the card is determined, is not without issues (see Section 2.4). Individuals with visible disabilities and families with better access to information and familiarity navigating local bureaucracies may be better placed to obtain a card and be over-represented in the sample.

Participants in supplementary qualitative interviews were purposively selected based on themes emerging from preliminary analysis of the quantitative data. While this allowed the exploration of specific items, such as home adjustments, the number of participants per selected topic area (see Section 2.6) was small. The overall number of qualitative participants was limited by budget and time constraints. In terms of general experiences, including comparison with preliminary interviews, little new information was generated in the last five (out of twenty-nine) interviews.

Capturing hypothetical costs is challenging in situations of limited information and a low or uneven availability of services. For example, expecting a parent to have complete knowledge of available health interventions and how these interventions may benefit their child is unrealistic. This can be compounded in situations where a child has had no specialist diagnosis or an incomplete diagnosis. Interviews with health professionals sought to offset this issue; however, there was a tendency for professionals to overestimate health-related costs (see Section 3.7). These professionals were mostly situated in the Metro Manila area. They should not be taken to be representative of the availability of services or the (additional) costs of accessing these services for households of children with disabilities in other areas. It should also be noted that costs vary over time and may increase or decrease with the age of the child.

## 5. Conclusions

Our findings show that children with disabilities and their families incur very significant extra costs. Using estimates from the asset index model, the child’s extra costs range from 40% to 80% depending on the severity of the disability. However, as the analysis of deprivations suggests, addressing the inequities behind these extra costs and high levels of unmet needs requires a range of policy instruments and programmatic interventions to be effective. More specifically, cash support alone cannot address disability-related expenses and inclusive policies in health and education are necessary to address the needs of children with disabilities and their families. Results based on the SOL method can differ significantly when using measures based on asset indexes and the number of deprivations. These measures provide different insights on the nature of disadvantage faced by persons with disabilities. Finally, while the measurement of the cost of disabilities is often computed at the household level, for poverty measurement it is important to compute these costs at the individual level using equivalence scales. This allows for the proper accounting of the impact of disability-related costs across households of different sizes and compositions.

## Figures and Tables

**Table 1:** Distribution of household types by presence of children with disabilities and average number of children with disabilities in the household.

Type of Household	Disability	No Disability	Average Number of Children with Functional Difficulties
Nuclear families	65.0	64.0	1.16
*Couple with 1 child*	*9.1*	*12.0*	*1.00*
*Couple with 2 children*	*17.8*	*18.3*	*1.11*
*Couple with 3 children*	*16.2*	*13.1*	*1.23*
*Couple with 4 or more children*	*13.6*	*11.5*	*1.32*
*Single parent with children*	*8.3*	*9.0*	*1.05*
Three-generation households	16.0	16.2	1.14
Other households with children	19.0	19.9	1.17
Total	100.0	100.0	1.16
Observations	1313	1440	

**Table 2:** Descriptive statistics of main variables by type of household and with/without children with disabilities.

Variable	Nuclear Families		Other Household Types	
	Disability	No Disability	Disability	No Disability
Consumption (log), mean	9.43	9.34	9.71	9.55
Standard deviation	0.60	0.56	0.62	0.62
Age of household head, mean	41.97	40.08	52.54	50.88
Standard deviation	8.58	9.83	14.03	14.77
Household size, mean	4.66	4.47	5.87	5.45
Standard deviation	1.50	1.46	2.03	2.04
Urban areas, mean	0.62	0.68	0.69	0.72
Standard deviation	0.45	0.44	0.42	0.42
Asset index, mean	−0.02	0.06	0.54	0.44
Standard deviation	1.39	1.23	1.38	1.29
Number of deprivations				
Three or more	10.7	6.4	4.1	4.6
Two	14.2	11.7	11.1	9.8
One	33.6	29.1	37.7	34.1
None	41.6	52.8	47.1	51.5
	100.0	100.0	100.0	100.0

**Table 3:** Regression results and estimated extra costs.

	Asset Index	Number of Deprivations
Variables		
Consumption (log)	1.151 ***	1.479 ***
Child with milder functional difficulties	−0.133 **	−0.395 ***
Child with severe functional difficulties	−0.201 ***	−0.806 ***
Age of household head	0.076 ***	0.144 ***
Age of household head squared	−0.001 ***	−0.001 ***
Couple with one child	0.177 **	0.356 **
Couple with three children	−0.188 ***	−0.386 ***
Couple with four or more children	−0.470 ***	−0.805 ***
One parent with children	0.008	−0.080
National Capital Region	0.006	0.114
Urban	0.444 ***	0.059
Visayas	−0.589 ***	−0.284
Mindanao	−0.498 ***	−0.569 ***
Number of observations	1775	1775
Model	OLS	Ordered logit
Pseudo R2/Adj. R2	0.442	0.110
**Proportional Extra Expenditure for Households with Children with Disabilities (Equation (3))**		
Milder functional difficulty	1.12 (exp(0.133/1.151))	1.31 (exp(0.395/1.479))
Moderate/severe functional difficulty	1.19 (exp(0.201/1.151))	1.72 (exp(0.806/1.479))

** is significant at the 95% confidence level; *** is significant at the 99% confidence level.

## Data Availability

Restrictions apply to the availability of data for ethical approval and contractual reasons. Queries should be addressed to the corresponding authors.

## Appendix A

**Table A1:** Asset index variables and coefficients.

Variable		First Principal Component Coefficient
Walls’ construction material:	Salvaged	−0.511
	Light	−0.126
	Strong	0.257
House tenure:	Without consent/papers	−0.369
	Rent free	−0.152
	Owned	0.107
Water access:	Un-improved	−0.206
	Improved	−0.039
	Piped in dwelling	0.145
Toilet type:	Un-improved	−0.541
	Improved	−0.370
	Flush to tank	0.041
	Flush to sewer	0.618
House squared meters		0.234
Car ownership:	No	−0.035
	Yes	0.773
Motorbike ownership:	No	−0.142
	Yes	0.221
Stove with oven/gas range:	No	−0.230
	Yes	0.259
Fridge/freezer ownership:	No	−0.248
	Yes	0.357
Personal computer ownership:	No	−0.083
	Yes	0.595
Smart phone ownership:	No	−0.411
	Yes	0.091
Television ownership:	No	−0.419
	Yes	0.164
